# A Randomized Clinical Trial Comparing the Effectiveness of Electroacupuncture versus Medium-Frequency Electrotherapy for Discogenic Sciatica

**DOI:** 10.1155/2017/9502718

**Published:** 2017-04-12

**Authors:** Xue Zhang, Yang Wang, Zhao Wang, Chao Wang, Wentao Ding, Zhishun Liu

**Affiliations:** ^1^Department of Acupuncture, South Area of Guang'anmen Hospital, China Academy of Chinese Medical Sciences, No. 138 Xingfeng North Street, Daxing District, Beijing 102618, China; ^2^Department of Acupuncture, Guang'anmen Hospital, China Academy of Chinese Medical Sciences, No. 5 Beixiange Street, Xicheng District, Beijing 100053, China

## Abstract

*Objective*. To investigate the short- and long-term effects of electroacupuncture (EA) compared with medium-frequency electrotherapy (MFE) on chronic discogenic sciatica.* Methods*. One hundred participants were randomized into two groups to receive EA (*n* = 50) or MFE (*n* = 50) for 4 weeks. A 28-week follow-up of the two groups was performed. The primary outcome measure was the average leg pain intensity. The secondary outcome measures were the low back pain intensity, Oswestry Disability Index (ODI), patient global impression (PGI), drug use frequency, and EA acceptance.* Results*. The mean changes in the average leg pain numerical rating scale (NRS) scores were 2.30 (1.86–2.57) and 1.06 (0.62–1.51) in the EA and MFE groups at week 4, respectively. The difference was significant (*P* < 0.001). The long-term follow-up resulted in significant differences. The average leg pain NRS scores decreased by 2.12 (1.70–2.53) and 0.36 (−0.05–0.78) from baseline in the EA and MFE groups, respectively, at week 28. However, low back pain intensity and PGI did not differ significantly at week 4. No serious adverse events occurred.* Conclusions*. EA showed greater short-term and long-term benefits for chronic discogenic sciatica than MFE, and the effect of EA was superior to that of MFE. The study findings warrant verification. This trial was registered under identifier ChiCTR-IPR-15006370.

## 1. Introduction

Sciatica is defined as radicular leg pain localized to the dermatological distribution of a pathologically affected nerve root. Almost all discogenic sciatica is induced by lumbar disc herniation (LDH) and may be accompanied by neurological deficits, such as leg pain, leg paresthesia, disability, and low back pain [1, 2]. The estimated prevalence of sciatica ranges from 1.2 to 43% in various regions [1]. Discogenic sciatica, which accounts for nearly 90% of sciatica, is a major cause of morbidity; moreover, it has a considerable impact on the economy due to both loss of work and the high costs of health care and societal support for the affected individual and his/her family [3, 4]. Current treatments for discogenic sciatica primarily include surgical and conservative treatments [5]. Although discectomy is a more effective treatment than other treatments for patients with severe discogenic sciatica, in patients with less severe symptoms, surgery or conservative treatments appear to be equally effective [6]. Discectomy should be avoided during initial treatment due to its high cost and its association with a higher incidence of postoperative complications, such as the loss of spine stability [7, 8] and extensive peridural fibrosis [9]. Conservative measures comprise the first-line treatment strategy for managing radicular pain due to disc herniation [10]. Regarding cost-effectiveness, the regimes that employ stepped approaches based on an initial treatment with conservative management have been recommended [11]. However, many conservative treatments have no explicit curative effect, such as benzodiazepines, corticosteroids, traction, and spinal manipulation, which may be ineffective or less effective [12]. Moreover, the long-term efficacy of analgesic drugs is not enduring, and intolerable side-effects, such as addiction, stomach ulcers, and constipation, occur frequently in patients with discogenic sciatica. Thus, based on recent information, the short- and long-term efficacy of conservative treatment should be evaluated [13].

Electroacupuncture (EA) has been used to treat sciatica for many decades in China. Several studies have reported that EA may effectively treat neuropathic pain and relieve sciatica symptoms [3, 4]. However, no clear clinical evidence exists to support the application of acupuncture or EA in the treatment of discogenic sciatica according to the guideline for the diagnosis and treatment of lumbar disc herniation [6]. Recently, two meta-analyses concerning sciatica treatment with acupuncture showed that previous studies on acupuncture were flawed and that the strength of the evidence was suboptimal; thus, studies of higher quality with longer-term follow-up are needed to clarify the long-term effect of acupuncture in sciatica patients [14, 15].

Compared with manual acupuncture, EA treatment is capable of increasing the stimulation frequency and intensity in a controlled and quantifiable manner [16]; moreover, its effect is superior to manual acupuncture for alleviating pain and improving paresthesia and dysfunction. Medium-frequency electrotherapy (MFE) is similar to transcutaneous electrical nerve stimulation (TENS) and may relieve pain and related symptoms. MFE works through electrostimulation of an electrode placed on the skin, and a battery powered device provided a small current to produce a tingling sensation. Several studies found that the effects obtained with 50 Hz EA were superior to those using 2 Hz EA [16, 17]. EA and MFE using the same frequency (50 Hz) at the same location were employed in another trial. The major difference between the two trial groups was the specific impact of needle penetration, with EA using needle penetration and MFE administered via nonpenetrating electrostimulation.

This study was a comparative trial that evaluated the effectiveness of EA versus MFE for the treatment of chronic discogenic sciatica; these treatments are the most frequently used treatments for this disease in China. We explored the ability of EA to alleviate leg pain, low back pain, and dysfunction at various evaluation time points, which included an assessment of the long-term efficacy of EA. We also assessed the patient global impression (PGI) and acceptance of EA compared with MFE and reports of adverse events.

## 2. Methods

### 2.1. Participants

The study commenced on May 28, 2015, and was completed by July 30, 2016, at the South Area of Guang'anmen Hospital, China Academy of Chinese Medical Sciences. Discogenic sciatica was diagnosed according to the criteria of the North American Spine Society [6]. The inclusion criteria were as follows: (1) individuals aged 18 to 70 years; (2) participants whose sciatica symptoms correlated with magnetic resonance imaging (MRI) or computed tomography (CT) findings of lumbar disc herniation; (3) participants whose symptoms of leg pain lasted more than 3 months; (4) participants who agreed to follow the trial protocol; and (5) participants who could complete the study treatment and assessments. The exclusion criteria were as follows: (1) participants with severe progressive neurological symptoms (e.g., cauda equina syndrome and progressive muscle weakness); (2) participants who had undergone surgery for lumbar disc herniation within 6 months; (3) participants with symptoms caused by conditions other than lumbar disc herniation that might lead to radiating pain in the leg; (4) participants with pain in both legs; (5) participants with cardiovascular, liver, kidney, or hematopoietic system diseases, mental health disorders, or cancer for whom EA might be inappropriate or unsafe; (6) participants who had received EA or electrotherapy within the past week; (7) women who were pregnant or lactating; (8) participants who were participating in other clinical trials; and (9) participants with a pacemaker, metal allergy, or severe fear of needles.

### 2.2. Study Design

This was a single-center, prospective, controlled, randomized trial conducted in patients with chronic discogenic sciatica. This trial was approved by the Ethics Committee of Guang'anmen Hospital of China Academy of Chinese Medical Sciences (approval number 2015EC042) on May 26, 2015, and was registered on May 7, 2015, at http://www.chictr.org.cn/ (ref. ChiCTR-IPR-15006370). Written informed consent was obtained from each participant or their legal representative. All participants were required to be able to understand written instructions and able to complete the pain assessment forms.

### 2.3. Randomization and Allocation Concealment

The randomization was performed by the Drug Clinical Trial Office affiliated with Guang'anmen Hospital using a computerized random number generator. Opaque, sealed envelopes were numbered consecutively, and all the sealed envelopes were maintained by a researcher who was not involved in the treatment procedure or data analysis. After informed consent was obtained, an envelope was opened by the researcher according to the patient's order of entry into the trial, and the assigned treatment was offered to the participant. The outcome assessors and statisticians were blinded to the allocation. Two copies of the envelopes were maintained to prevent the researchers from deviating from the randomization.

### 2.4. Intervention

The treatments were initiated one week after participant randomization. All participants received health education on sciatica, such as using a hard bed and losing weight. During the trial, the use of analgesic drugs or other treatments was not permitted. The details of prior drug use (including dose and time) were recorded in the medication record form. Huatuo Brand stainless steel needles (0.3 × 100 mm, Suzhou Medical Appliance Factory in China, CL) and a G6805-2 electric stimulator (Shanghai Huayi Medical Instrument in China Co., Ltd.) were used in the EA group, and the Quanrikang type J48A computerized intermediate-frequency therapy apparatus (Beijing Huayi New Technical Institute in China) was used in the MFE (control) group. The acupuncture procedures were performed in accordance with the Standards for Reporting Interventions in Clinical Trials of Acupuncture (STRICTA) guidelines [18]. EA was performed by a trained clinician with more than 2 years of experience with acupuncture manipulation. The acupuncture regimen was based on our own pilot trial and specialist consensus. The acupoints of the affected side (DaChangShu, BL25) and the bilateral JiaJi (Ex-B2) corresponding to LDH were included in the EA group. The DaChangShu (BL25) acupoint was located according to the World Health Organization Standardized Acupuncture Point Location [19]; JiaJi (Ex-B2) is located in the lumbar region 0.5 inches lateral to the posterior median line. After the participants assumed a prone position, the needle was vertically inserted rapidly into the JiaJi (Ex-B2) points. Then, the needle was inserted to a depth of approximately 1.5 inches. The participants were expected to experience soreness and distension transmitted to the leg. The needle was inserted straight into the DaChangShu on the BL25 point to a depth of 3 inches; then, the acupuncturist manipulated the needle with a lifting, thrusting, and twirling maneuver until feelings of soreness and distension were felt and radiated to the hips and lower limbs. The electric apparatus was applied to the JiaJi (Ex-B2) and DaChangShu (BL25) acupoints with a dilatational wave using a 50 Hz frequency and a comfortably tolerated maximum current intensity.

Participants assigned to the control group received MFE, which was administered by an experienced therapist different from the one delivering the EA. The acupoints and frequencies used in the MFE group were the same as those used in the EA group. After two pairs of 107 × 72 mm electrodes were placed on the acupoints, the MFE apparatus was turned on and muscle contractions were observed under the energizing electrode. The intensity was adjusted to the maximum current intensity tolerable at a comfortable level. The treatments in both groups were performed once daily for 5 sessions/week for the first 2 weeks and followed by 3 sessions/week for the following 2 weeks, with each session lasting 20 minutes.

### 2.5. Data Collection

The data in the trial were obtained from the case report forms recorded by the investigator. The participants' demographic, clinical, and radiological characteristics were recorded. The diagnosis of lumbar disc herniation was confirmed after a review of the patient's MRI or CT scan by two experienced musculoskeletal radiologists. Additionally, the diagnosis of discogenic sciatica was confirmed after a clinical examination by a consultant orthopedic physician. Investigators entered the collected data into the case report forms. At baseline and during the treatment period, the forms were completed by the participants under the guidance of a full-time staff member. During the follow-up period (16th and 28th weeks), the participants answered the questionnaire by phone.

### 2.6. Clinical Assessments

The primary outcome was the change from baseline in the average leg pain numerical rating scale (NRS) score at week 4 [20]. The secondary outcomes included average leg pain intensity at weeks 1, 2, 3, 16, and 28; low back pain intensity at weeks 2, 4, 16, and 28; Oswestry Disability Index (ODI) questionnaire results at weeks 2, 4, 16, and 28; PGI of improvement at weeks 2 and 4; drug use frequency at weeks 2 and 4; and EA acceptance evaluation at week 4. Adverse events were monitored and documented during the treatment and follow-up periods based on the investigator's inquiry and reports by the participants themselves.

#### 2.6.1. Primary Outcome Measure

The change from baseline in the average leg pain NRS score was measured using an 11-point numerical rating scale assessing leg pain, with 0 representing no pain and 10 representing the most severe pain. Participants were asked to rate their average leg pain intensity over the prior 24 hours. The average leg pain NRS score at week 4 was equal to the mean value of the NRS scores obtained at the three treatment sessions during the 4th week.

#### 2.6.2. Secondary Outcome Measures

The following secondary outcome measures were determined. (1) The average leg pain intensity at other time points was measured by the NRS. The methods used to measure the secondary outcomes were the same as those used to measure the primary outcome except for the evaluation point. (2) Low back pain intensity was measured using an 11-point NRS. Participants rated their low back pain over the prior 24 hours with a pain NRS. The low back pain NRS score at the time of evaluation was equal to the mean value of the NRS scores in the previous 24 hours. (3) The ODI comprises 10 questions concerning the intensity of pain and daily activities [21]. Each item contains 6 options. A higher score change in the ODI from baseline indicated more serious dysfunction. (4) The PGI improvement score was used to evaluate the improvement in pain and functional disability, and the improvement reported by patients was assessed using a 7-point scale (1 represents greatly improved and 7 represents marked worsening) [22]. (5) The frequency of drug use was recorded. The patients' use of medications or nonprescription drugs during the trial was evaluated using a questionnaire to assess the influence of drugs. (6) To investigate which treatment was preferred, EA or MFE acceptance was assessed at week 4. A 4-point scale was used, with 1 representing “very difficult to accept” and 4 representing “very easy to accept.” (7) Adverse events were assessed using a questionnaire at the end of treatment and active reporting by the participants during treatment.

### 2.7. Sample Size and Statistical Analysis

The sample size calculation was based on the mean value of the leg pain intensity NRS score. According to our pilot trial, the decreases in the mean value of the leg pain intensity NRS scores in the EA and MFE groups at week 4 were 3.41 ± 3.46 and 1.57 ± 1.24, respectively. Our pilot study was an independent study conducted by our research team before this study, with no crossover participants between the previous study and the current study. We used PASS Version 11.0 (International Business Machines Corporation, China) software to calculate a sample size of 50 for each group to provide 90% power to detect a difference of 1.8 between the groups with a two-sided 5% level of significance, allowing for a 20% dropout rate and with the participants receiving the treatments and completing the follow-up.

The statistical analysis was performed using SPSS Version 22.0 (International Business Machines Corporation, China) software. Two-sided tests were used for all statistical analyses. The level of significance was established at 0.05. All patients who accepted randomization were included in the analysis. All data collected from the participants were included in the statistical analysis, and missing data were replaced by the last observed value. However, the outcomes for which no data except for the baseline assessment data were available were not included in the final analysis. The 100 participants included at least 1 treatment session. Thus, we analyzed the data of all the participants as the primary outcome, which was measured after the first treatment session. However, the secondary outcomes were evaluated at week 2, and 13 participants dropped out before week 2 without any data after treatment except for leg pain NRS scores. So the 13 participants were not included in the statistical analysis of secondary outcomes. Continuous data were represented by means and standard deviations (SD) if the data were normally distributed or by the medians and interquartile ranges if the data were skewed, or by means and 95% confidence intervals (CIs); categorical data were represented by percentages or 95% CIs. For comparisons with baseline data, a paired *t*-test was used for continuous data and a nonparametric test was used for categorical data. To compare the two independent samples, *T* tests or Mann–Whitney *U* tests were used to compare continuous variables, and chi-square tests or Fisher's exact tests were used to compare categorical variables, as appropriate. A repeated measures analysis of variance or nonparametric test was used to compare differences in data between the groups at multiple time points.

## 3. Results

### 3.1. Recruitment

**Figure 1:**
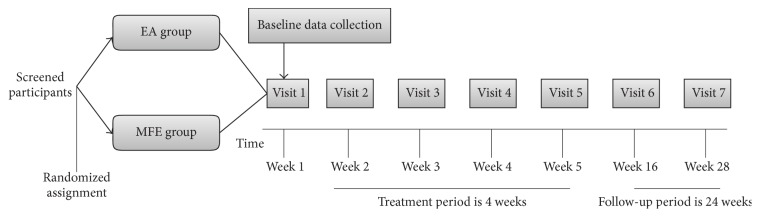
Time frame of each period.  Figure 1 shows the time frame of baseline period, treatment period, and follow-up period.

**Figure 2:**
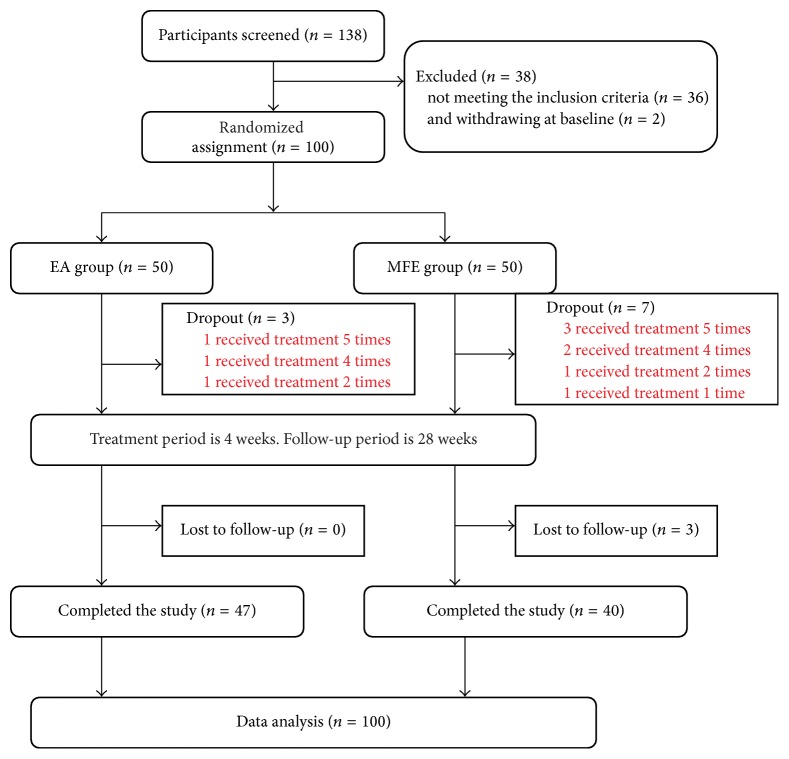
Study flow diagram.

A total of 138 participants with chronic sciatica due to lumbar disc protrusion were screened, among whom 36 were rejected due to the exclusion criteria and 2 withdrew from the study. Therefore, 100 eligible patients were randomly assigned to the experimental (EA) group (*n* = 50) or the control (MFE) group (*n* = 50) at a ratio of 1 : 1. Eight participants withdrew from the study during the course of treatment due to the presence of aggravating symptoms, 1 participant exited the study due to travel, 1 participant withdrew due to an unsatisfactory curative effect, and 3 participants were lost to follow-up. In the dropout participants, no additional data except for the leg pain NRS scores were available because the evaluation period was not reached. According to the principle of ITT analysis, we analyzed the data of all 100 subjects for the leg pain NRS scores and then performed a sensitivity analysis of these 13 subjects to verify the reliability of the results. Details are provided in Figures 1 and 2.

### 3.2. Characteristics of the Participants

Table 1 shows the baseline data of the 100 participants. The mean age of all patients was 52.67 ± 12.72 years. The mean duration was 48 (12–120) months. The duration of 2 participants in the EA group was one month, and the duration of 1 participant was one month in the MFE group. The baseline demographics, body measurement data, and baseline outcomes are listed in Table 1. No significant differences in baseline demographics and clinical characteristics were observed (Table 1).

### 3.3. Primary Outcome

The decrease in the leg pain NRS scores from baseline to week 4 differed significantly between the EA group (*n* = 50) and the MFE group (*n* = 50) (*P* < 0.001). As shown in Table 2, the mean change from baseline to the 4th week in the average leg pain intensity NRS score was 2.30 (1.86–2.75) in the EA group and 1.06 (0.62–1.51) in the MFE group. At four weeks, the two groups both exhibited significantly greater reductions in NRS scores compared with baseline; however, the EA group showed a more significant decrease than the MFE group (Table 2).

### 3.4. Secondary Outcomes

**Figure 3:**
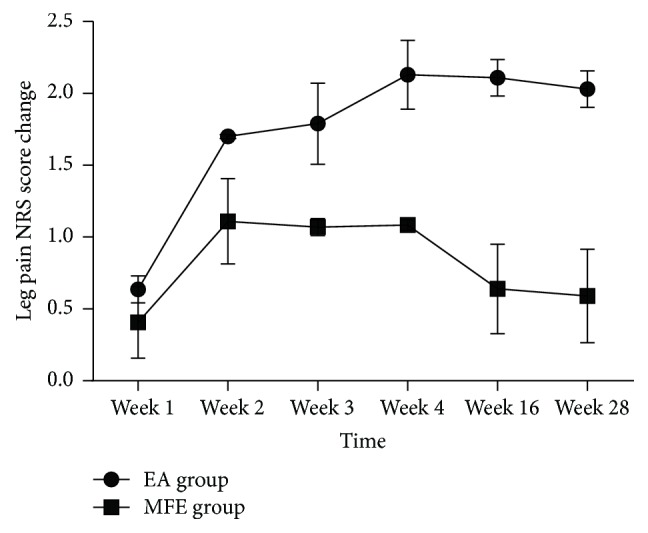
Change of leg pain score in two groups.

EA showed a more significant improvement in the leg pain scores at all the evaluation points compared with that observed in the MFE group (*P* < 0.001) (Figure 3 and Table 2). The EA group showed a significant decrease compared to the baseline in the leg pain, low back pain, and ODI scores at weeks 2, 4, 16, and 28 (all *P* < 0.05). Conversely, the MFE group did not show a significant improvement compared to the baseline in the low back pain score at weeks 16 and 28 (all *P* = 0.096). Significant reductions in the leg pain and ODI questionnaire scores were detected in the EA group at multiple time points compared with the MFE group (all *P* < 0.05). The EA group exhibited greater improvement. However, a negligible change was detected at multiple time points in the low back pain score and PGI between the two groups (all *P* > 0.05). Furthermore, no significant difference was detected in the frequency of drug use between the two groups at weeks 2 and 4 (all *P* > 0.05) in our trial. Consequently, an EA or MFE acceptance assessment administered after 4 weeks of intervention showed that EA was accepted as readily as MFE with no significant differences between the two groups (*P* = 0.055). The corresponding data are shown in Tables 2 and 3.

A sensitivity analysis was performed based on the leg pain NRS score. We excluded 13 participants who received fewer treatment sessions (less than 10) and analyzed the data of the remaining 87 participants. This sensitivity analysis result showed that our original results were stable and reliable.

### 3.5. Adverse Events

No serious adverse events occurred in either group. One participant (2%) in the experimental group developed a subcutaneous hematoma. Two participants (4%) in the MFE group reported skin redness and itching. All adverse events disappeared without additional intervention.

## 4. Discussion

The results of this trial showed significant differences in the change in the leg pain NRS and ODI questionnaire scores in the EA group compared with those in the MFE group in the short-term treatment period and long-term follow-up. However, the EA group did not show a greater decrease in low back pain scores and PGI compared with the MFE group. These changes indicated that the effect of EA was superior to the effect of MFE in improving leg pain and dysfunction, whereas the effect of EA was not superior to that of MFE in relieving low back pain and systemic symptoms.

The leg pain NRS score showed a significant difference compared with the MFE group at week 4: a mean difference of 1.24 points was detected between the two groups. On average, a reduction of approximately 2–3.5 points in the NRS score represents a minimal clinically important difference (MCID) for acute and chronic pain [23, 24]. The change in the leg pain NRS score in the EA group at week 4 did not show a clinically important significant difference compared with the MFE group. However, our control group was not a placebo but a positive treatment. An effect size of 1.24 is generally considered as the large effect. The MCID of the ODI score ranged from 4 to 16 points [23], and the decline of the ODI score in the EA group reached the MCID criterion with a mean reduction of 5.69 compared with the MFE group. The results implied that the clinical effect of EA appears superior to the effect of MFE in improving dysfunction caused by sciatica. However, low back pain did not show a significant and clinically important difference, with a mean reduction in the NRS score of 0.58 at week 4 compared with the MFE group. It may be associated with a better response to pain around the electrodes by MFE. In our study, a long-term follow-up was performed. At week 28, the MFE group did not show significantly decreased leg pain compared to the baseline, whereas the EA group showed significantly decreased leg pain compared to the baseline. The difference between the two groups was significant. The results implied that the effect of EA but not MFE lasted at least 28 weeks. The low back pain and ODI scores also indicated that the long-term effects of EA were superior to those of MFE because the effects of EA persisted after the discontinuation of treatment.

In our trial, the leg pain NRS score was reduced by 49% compared with the baseline in the EA group at week 4; however, a greater increase in the response rate (69%) was reported in a trial comparing EA with TENS for sciatica [25] during the treatment period. Another trial conducted in China demonstrated [26] that the decrease in the mean value of the leg pain intensity NRS score in the EA group was 4.65 ± 6.37 at week 4, which was higher than the value of 2.30 (1.86–2.75) obtained in our trial at the same time point. In a pilot trial comparing EA with physical therapy for symptomatic lumbar spinal stenosis (LSS) [27], pain in the back and leg showed small improvements at 3 months. However, the ODI scores were different from the scores obtained in our study. No significant differences between the ODI scores of the two groups were observed at the 3-month follow-up time point in the study. The differences between the results of the two studies might be explained by the use of different acupoints, needling depth, manipulation methods, EA parameters, number and frequency, training and clinical experience level of the practitioners, missing data, and sample size.

Very few participants in either group took analgesics during the trial, and only anti-inflammatory drugs were used. This result might indicate that most of the participants believed that the analgesics would not alleviate pain and were concerned about adverse events. Most participants expected that EA or MFE would be beneficial and were aware that these techniques are relatively safe. According to the PGI, the participants perceived no difference between EA and MFE. Approximately 87.2% of the participants in the EA group reported that they were aided by EA at the 4th week, which was similar to the 83.5% of participants in the MFE group. The treatment acceptance assessment showed that none of the participants considered either treatment difficult to accept. Furthermore, 70.2% of the participants in the EA group reported that EA was easy or very easy to accept, similar to 72.5% of the participants in the MFE group. These results indicated that EA and MFE were both easy to accept and popular in China.

Leg pain is a typical symptom in sciatica patients, and the leg pain intensity NRS score reflects the improvement in this symptom in these patients. The leg pain NRS score may reasonably be used for the primary measurement of the therapeutic effect. Because studies have shown that most acupuncture therapy for sciatica lasts 1 to 4 weeks [25], we selected the change in the average leg pain intensity NRS score from baseline to the 4th week as the main measurement. In the previous literature, although primary outcome was generally measured at a certain time point, the average score reflected the average level of pain during the last week, which was thus more meaningful than other methods of measuring single time point due to recurrence of sciatica. The control group underwent MFE, which exerts its effect via the stimulation or activation of physiological events by applying energy, thereby producing therapeutic benefits that facilitate pain relief [28]. Mechanisms leading to pain relief may be due to a variety of peripheral effects of control activity, on the spinal and spinal nervous system. The comparison between EA and MFE may reveal differences in response to needle penetration using the same electrostimulation. Because the stimulation parameters, particularly the frequency, are important factors that affect the outcome and because the effect of medium-frequency electrotherapy is better than the effect of low frequency electrotherapy [15, 16], we used the same medium frequency and location in the study to ensure that the two groups were comparable.

Many studies have investigated the mechanism of EA. EA has been reported to relieve the symptoms of sciatica and increase the pain threshold in humans [29]. Several previous studies showed that EA inhibited the primary afferent transmission of neuropathic pain [30] and that deep EA stimulation improved the pathological changes and function of the injured sciatic nerve in rats [31]. Other studies have suggested that descending inhibitory control, changes in nerve blood flow, or the inhibition of activity by nerve endings may be involved in the mechanism associated with the efficacy of EA [32]. Long-lasting alleviation of pain has been suggested to be closely related to the muscle tension improvement provided by EA [33]. A meta-analysis of patients with chronic pain showed that approximately 90% of the benefit of acupuncture was sustained at 12 months [16]. The reason for the cumulative and sustained effects of acupuncture may be associated with the brain response and the cumulative duration of acupuncture stimulation [34].

This trial has several limitations. First, the participants and acupuncturists could not be blinded due to the significant difference between the two treatments. However, we followed rigorous quality control procedures in other aspects of the methodology. For example, a strict randomization and allocation concealment protocol was adopted. The outcome assessors and statisticians were blinded to the allocation. Second, some of the outcome measures of the trial were subjective. To address subjectivity, a short training session for the patients on the outcome reporting was held before they began the trial, and all subjective outcomes were based on the patient self-report forms. Third, we did not include a placebo control in the present preliminary study because several sham acupuncture randomized controlled trials (RCTs) have been performed to study acupuncture therapy in patients with sciatica [35]. We considered that the use of a placebo did not provide sufficient sensitivity and may not have met ethical guidelines. Fourth, because we did not explore the effect of EA on various degrees of pain severity, which degree of sciatica was most sensitive to EA was unclear. Subgroup analyses based on sciatica severity should be performed in a future multicenter, large-sample, randomized controlled study.

## 5. Conclusions

This randomized controlled clinical trial demonstrated that the short-term and long-term effectiveness of EA were superior to those of MFE in improving the symptoms of leg pain and dysfunction caused by chronic discogenic sciatica; moreover, the long-term effect of EA was superior to that of MFE in improving low back pain. The results also suggested that the effect of EA but not MFE lasted at least 28 weeks. No serious adverse events occurred in either group. Further studies are needed to examine the effectiveness of EA relative to various physical therapy methods for patients with discogenic sciatica.

## Figures and Tables

**Table 1:** Baseline demographic and clinical characteristics of the study population.

Characteristics	EA group (*n* = 50)	MFE group (*n* = 50)	*P* value
Age, years^†^	54.26 ± 12.39	51.08 ± 12.98	0.213^*∗*^
Sex, M/F (%)	21.3/78.7	22.5/77.5	0.467^&^
Body mass index, Kg/m^2^^†^	25.47 ± 3.25	24.78 ± 3.54	0.311^*∗*^
Duration of disease, months^‡^	60 (12–120)	48 (15–120)	0.613^#^
Leg pain NRS^†^	4.66 ± 1.88	4.35 ± 1.28	0.343^*∗*^
Low back pain NRS^†^	3.94 ± 2.41	3.68 ± 2.28	0.582^*∗*^
ODI questionnaire scores^†^	37.77 ± 16.79	35.00 ± 13.42	0.363^*∗*^
Frequency of analgesics (%)			0.806^&^
0 times/week	38 (76)	39 (78)	
1–3 times/week	4 (8)	6 (12)	
4–6 times/week	3 (6)	1 (2)	
7–9 times/week	2 (4)	2 (4)	
≥10 times/week	3 (6)	2 (4)	

EA: electroacupuncture; MFE: medium-frequency electrotherapy; ^†^mean (standard deviation).

NRS: numerical rating scale; ODI: Oswestry Disability Index; ^&^chi-square test.

^*∗*^Unpaired *t*-test; ^#^Mann–Whitney *U* test; ^‡^median (interquartile ranges).

**Table 2:** Changes from baseline in primary outcomes.

Leg pain NRS	EA group (*N* = 50)	MFE group (*N* = 50)	Differences	*P* value^&^
Time	Mean (95% CI)	Mean (95% CI)	Mean (95% CI)	
Week 1	0.57 (0.39–0.75)^*∗*^	0.23 (0.05–0.41)^*∗*^	0.34 (0.11–0.57)	0.010^‽^
Week 2	1.71 (1.28–2.13)^*∗*^	0.90 (0.47–1.32)^*∗*^	0.81 (0.25–1.37)	0.008^‽^
Week 3	1.99 (1.60–2.37)^*∗*^	1.02 (0.63–1.41)^*∗*^	0.97 (0.41–1.52)	0.001^‽^
Week 4	2.30 (1.86–2.75)^*∗*^	1.06 (0.62–1.51)^*∗*^	1.24 (0.59–1.88)	<0.001^‽^
Week 16	2.20 (1.76–2.63)^*∗*^	0.42 (−0.01–0.86)^*∗*^	1.78 (1.19–2.35)	<0.001^‽^
Week 28	2.12 (1.70–2.53)^*∗*^	0.36 (−0.05–0.78)^*∗*^	1.76 (1.18–2.32)	<0.001^‽^
Total time points of weeks 1–28				<0.001^§^

NRS: numerical rating scale; MFE: medium-frequency electrotherapy; ^‽^*T*-tests.

EA: electroacupuncture; CI: confidence interval; ^*∗*^*P* value compared to baseline < 0.05.

& indicates *P* value for the between-group comparison; ^§^a repeated measures analysis of variance.

**Table 3:** Secondary outcomes of the interventions.

Variable	EA group (*N* = 47)	MFE group (*N* = 40)	*P* value^&^
	Mean (95% CI)	Mean (95% CI)	
*Low back pain NRS*			
Week 2, change from baseline	2.12 (1.59–2.66)^*∗*^	1.47 (0.89–2.05)^*∗*^	0.104^‽^
Week 4, change from baseline	2.23 (1.68–2.78)^*∗*^	1.65 (1.05–2.25)^*∗*^	0.158^‽^
Week 16, change from baseline	2.00 (1.54–2.46)^*∗*^	0.40 (−0.09–0.89)^*∗∗*^	<0.001^‽^
Week 28, change from baseline	2.00 (1.55–2.45)^*∗*^	0.40 (−0.08–0.88)^*∗∗*^	<0.001^‽^
Total time points of weeks 1–28			0.072^§^
*ODI questionnaire*			
Week 2, change from baseline	6.79 (4.35–9.23)^*∗*^	6.49 (3.85–9.13)^*∗*^	0.868^‽^
Week 4, change from baseline	12.34 (9.39–15.30)^*∗*^	6.65 (3.44–9.85)^*∗*^	0.011^‽^
Week 16, change from baseline	11.29 (8.55–14.04)^*∗*^	3.63 (0.66–6.60)^*∗*^	<0.001^‽^
Week 28, change from baseline	10.95 (8.42–13.47)^*∗*^	1.87 (−0.86–4.61)^*∗*^	<0.001^‽^
Total time points of weeks 1–28			0.001^§^

*Patient global impression, number (%)*			
Week 2	Great: 3 (6.4%), moderate: 15 (31.9%) little: 24 (51.1%), no: 5 (10.6%)	Great: 2 (5%), moderate: 9 (22.5%), little: 22 (55.0%), no: 7 (17.5%)	0.665^#^
Week 4	Great: 7 (14.9%), moderate: 23 (48.9%), little: 11 (23.4%), no: 6 (12.8%)	Great: 2 (5%), moderate: 13 (32.5%) little: 18 (45.0%), no: 7 (17.5%)	0.073^#^

*Drug use frequency, number (%)*			
Week 2	0 times: 42 (89.4%), 1–3 times: 3 (6.4%) 4–6 times: 1 (2.1%), 7–9 times: 1 (2.1%)	0 times: 33 (82.5%), 1–3 times: 3 (7.5%) 4–6 times: 4 (10.0%), 7–9 times: 0,	0.28^#^
Week 4	0 times: 44 (93.6%), 1–3 times: 2 (4.3%) 4–6: times 1 (2.1%), 7–9 times: 0	0 times: 36 (90.0%), 1–3 times: 2 (5.0%) 4–6 times: 2 (5.0%), 7–9 times: 0	0.749^#^

*Treatment acceptance assessment, number (%)*	Little difficult: 0, Moderate: 14 (29.8%), Easy: 16 (34%), Very easy: 17 (36.2%)	Little difficult: 0, Moderate: 11 (27.5%), Easy: 18 (45.0%), Very easy: 11 (27.5%)	0.055^#^

NRS: numerical rating scale; EA: electroacupuncture; MFE: medium-frequency electrotherapy; CI: confidence intervals.

^#^Chi-squared test; ^*∗∗*^*P* value compared to baseline > 0.05; ^*∗*^*P* value compared to baseline < 0.05.

^‽^ *T*-tests; & indicates *P* value for the between-group comparison; ^§^a repeated measures analysis of variance secondary outcomes analysis was done only with complete cases.
